# The Caribbean Community Clinical Oncology Workforce: Analyzing Where We Are Today and Projecting for Tomorrow

**DOI:** 10.1155/2018/7286281

**Published:** 2018-04-15

**Authors:** Kellie Alleyne-Mike

**Affiliations:** National Radiotherapy Centre, St. James, Port of Spain, Trinidad, Trinidad and Tobago

## Abstract

**Purpose:**

To analyze the current physician clinical oncological workforce within the CARICOM full member states with an aim to make recommendations for building capacity.

**Methods:**

A questionnaire was prepared and emailed to professionals working in oncology in 14 CARICOM full member countries. It was designed to identify the number of specialists providing hematology, medical oncology, and radiotherapy services.

**Results:**

Ten countries (71.4%) supplied information. Oncology services were insufficient in the majority of countries. Hematology proved to be the most adequately staffed with six countries (60%) having the recommended number of specialists. Medical oncology services were deficient in five countries (50%). Radiation oncology services were the most limited with nine countries (90%) unable to provide the required quota of specialists. The majority of the workforce consisted of nonnationals (55%). The remaining practitioners were nationals, and of these 50% were regionally trained. Oncological care was primarily offered within the public sector.

**Conclusion:**

Oncological staffing within the CARICOM full member states is insufficient to meet the demands of the current population. Encouraging training through locoregional or international programs is key to obtaining the numbers required. Cancer registries will help provide data to influence public policy and improve the oncological healthcare system.

## 1. Introduction

Regional cancer data for the Caribbean and Latin American region has been limited when compared to statistics emanating from North America, Europe, and Asia. The developing nations have relatively high incidences of cancer and increased mortality rates and are often less equipped to manage the disease burden optimally [1]. The Caribbean is often linked with Latin America in many studies, and thus data unique to that region have been difficult to isolate from the collective. This paper serves as a preliminary review of the oncology workforce in the region with its focus being on the full member states of the CARICOM nations. CARICOM comprises developing states and was founded in 1973. It consists of fifteen member states and five associate members with a number of services including resource mobilization to support regional integration. In the context of identifying resource limitations and methods for optimizing the use of existing services, these organizations are critical.

## 2. Materials and Methods

A questionnaire was prepared with questions relating to the available core oncological services being provided in each country. The survey was conducted between September 2016 and February 2017. Participants were primarily identified from previous attendees to the biannual conference hosted by the Caribbean Association of Oncology and Hematology. Participants were medical professionals or allied healthcare professionals.

Respondents were emailed the questionnaire which included instructions for completion. Follow-up emails were conducted if further information or clarification was necessary. Questions which referred to the human resource capacity were limited to the following groupings: medical oncologists, radiation oncologists, clinical oncologists (trained in both medical and radiation oncology), hematologists, and hemato-oncologists (instructed in both blood-based and solid tumor management). Questionnaires were sent to personnel working in oncology in fourteen CARICOM full member countries, namely, Antigua and Barbuda, Bahamas, Barbados, Belize, Trinidad and Tobago, Dominica, Grenada, Guyana, Haiti, Jamaica, St. Kitts and Nevis, St. Lucia, St. Vincent and the Grenadines, and Suriname. Montserrat was excluded from the primary analysis as this country is a British overseas territory and thus could not reasonably be compared to the remaining CARICOM members.

The specialists identified had to be actively in clinical practice. Recommendations for specialists numbers in medical oncology were taken from estimations by an American Society of Clinical Oncology (ASCO) survey estimate which suggested that 1.8 medical oncologists were required per 100,000 inhabitants [2]. The projections for radiation oncologists were made using guidance from the International Atomic Energy Agency (IAEA) which gauged the requirement for radiation oncologists as 1 per 100,000 inhabitants [3]. Information regarding the number of teletherapy units in each country was sourced from the IAEA Directory of Radiotherapy Centres (DIRAC) [4] and the recommendations were based on IAEA guidelines which suggested 1 machine per 180,00 inhabitants [3]. The numbers needed for hematology were sourced from an article review of an American Society of Hematology (ASH) panel discussion on that topic and proposed that 5 hematologists per 1,000,000 inhabitants would adequately meet the demand [5]. Information on population size and population density was collected using data obtained from the World Bank website [6]. Statistics on cancer incidence and mortality were sourced from GLOBOCAN 2012. The estimated staff numbers were rounded up to the nearest integer. Tables and graphs were prepared using Microsoft Word.

## 3. Results

Complete information was received from 71.4% of the fourteen countries except for The Bahamas, Guyana, St. Lucia, and Haiti which were thus excluded. All remaining countries had some level of oncology support. In St. Kitts and Nevis, hematology support is offered by a visiting specialist residing in the United States Virgin Islands. In the case of Belize a national, who now lives in the United States, offers part-time support. In the other nations, there was at least one professional who resided in the country while providing support.

**Figure 1:**
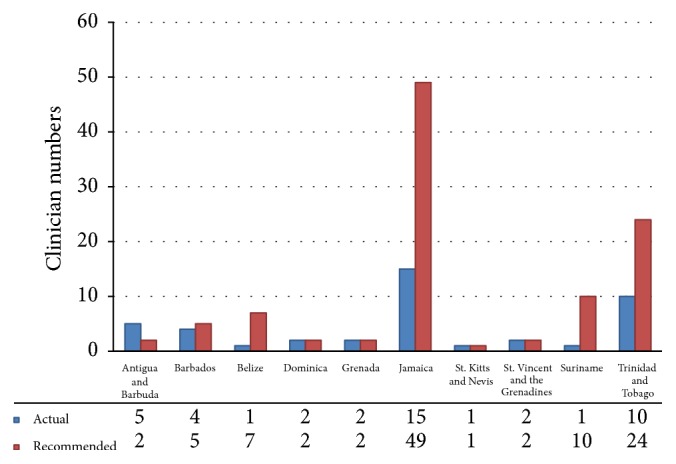
List of existing medical oncologists and required numbers based on a survey by the American Society of Clinical Oncologists (1.8 oncologists were required per 100,000 inhabitants).

**Figure 2:**
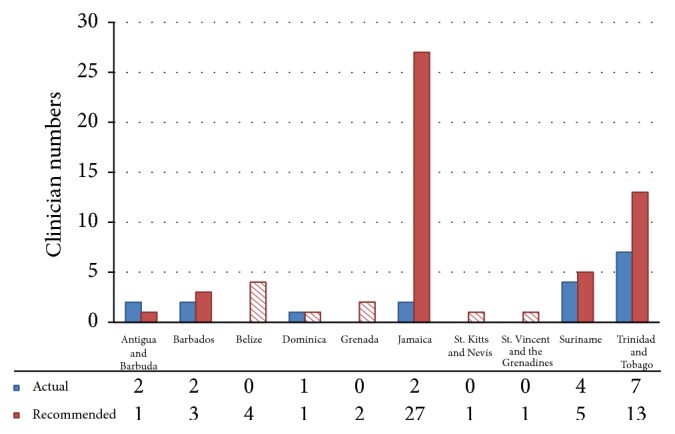
List of existing radiation oncologists and required numbers based on recommendations by the International Atomic Energy Agency (1 per 100,000 inhabitants).* Note*. Patterned bars indicate countries in which radiation therapy is not offered due to the lack of equipment.

**Figure 3:**
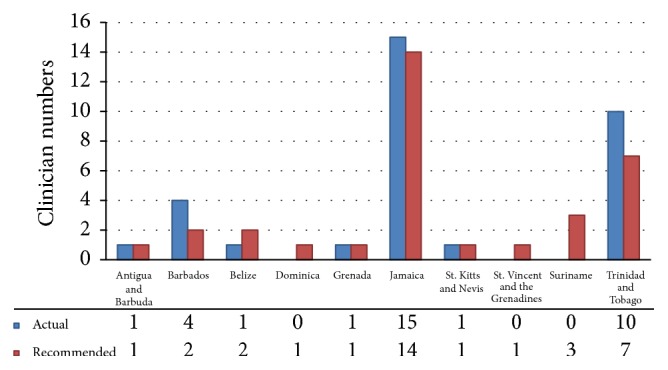
List of existing hematologists and required numbers based on recommendations from the American Society of Hematology (5 hematologists per 1,000,000 inhabitants).

Figures 1, 2, and 3 illustrate the number of doctors in each country who are capable of providing specialist attention in each of the three chosen specialty fields: medical oncology, radiation oncology, and hematology, respectively. The physician tally for each bar graph allowed for the recognition of dual specialties in separate graphs where physicians were dually trained.

In the case of medical oncology (Figure 1), Antigua and Barbuda and St. Kitts and Nevis were the only countries which appeared to surpass the required number of specialists. Dominica, St. Vincent and the Grenadines, and Grenada each met their recommended numbers while the remaining five islands (accounting for 50% of the studied group) appeared to have an inadequate supply of specialists. Jamaica had the greatest need with only 30.6% of the medical oncologists required being available. A total of four countries (Jamaica, Trinidad and Tobago, Suriname, and Belize) had less than half of the recommended staff to fulfill the medical oncology needs of the populations which they serve. In summary, there were five countries which possessed an adequate number of specialists accounting for 50% of this grouping.

In the case of radiation oncology services (Figure 2), five nations (denoted by patterned bars) did not have machines available to deliver therapeutic radiation. These included St. Vincent and the Grenadines, Dominica, St. Kitts and Nevis, Grenada, and Belize. The remaining countries offered radiation therapy but had inadequate staffing (except for Antigua and Barbuda). Suriname with 80% capacity and Barbados with 66.7% were the closest in meeting their recommended quotas. Jamaica had the least radiation oncology staffing for its population with only 7.4% of the necessary staff. In the case of Antigua and Barbuda, the available staffing again surpassed the required numbers. Thus nine countries, accounting for 90% of the grouping, had suboptimal human resource capacity. Among the countries providing radiation therapy services, a further analysis of the necessary equipment was undertaken. Based on IAEA recommendations on the number of radiation therapy machines per population size, it was found that the existing numbers were also inadequate to sustain the demand (Table 1). It should be noted that the IAEA document referred to the availability of a Linear Accelerator (LINAC) as the machine of choice for external beam radiation therapy. However, in some of the islands Cobalt-60 machines were used. Considering the limited resource setting these were included in the analysis and estimations for each country.

When analyzing hematology services (Figure 3), the quota within the Caribbean was more favorable. Jamaica, Barbados, and Trinidad and Tobago were the most equipped to meet the human resource demand for specialist services. They each appeared to surpass their recommended quotas by one individual (7.1%) in the case of Jamaica, two in the case of Barbados (100%), and three (42.9%) in the case of Trinidad and Tobago. Three other countries (Antigua and Barbuda, Grenada, and St. Kitts and Nevis) had the necessary staffing. The remaining four countries (40%) did not have the required staff to meet the imposed demands. Thus 60% of all countries possessed adequate hematological support.

**Figure 4:**
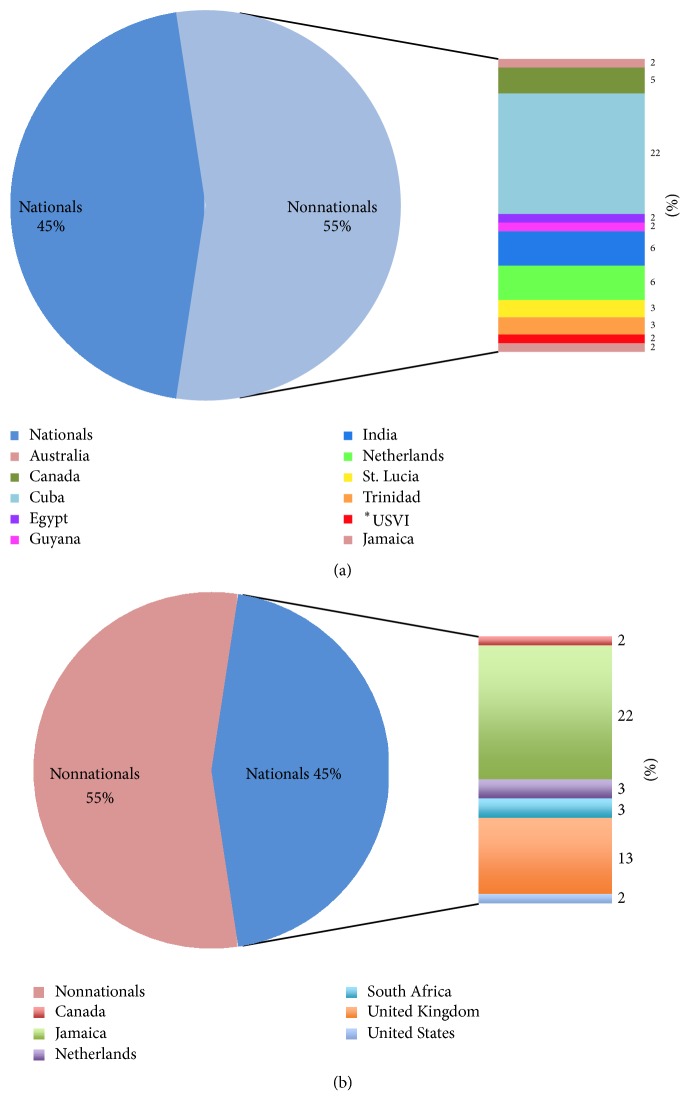
This illustrates the distribution of (a) nonnationals (based on the country of origin) to (b) nationals (based on the country of training). ^*∗*^USVI: United States Virgin Islands.

Figure 4 illustrates the distribution of nationals (based on the country of training) and nonnationals (based on the country of origin). There was a greater percentage of nonnationals (55%) to nationals (45%). The latter of the two groupings predominantly sourced local training in Jamaica (22%) where a specialist program was available for hematology and oncology. The United Kingdom was next in line with 13% of professionals accessing training in that country. When reviewing nonnationals, the majority were from Cuba (22%) followed by the Netherlands and India (each with 6% of the grouping).

**Figure 5:**
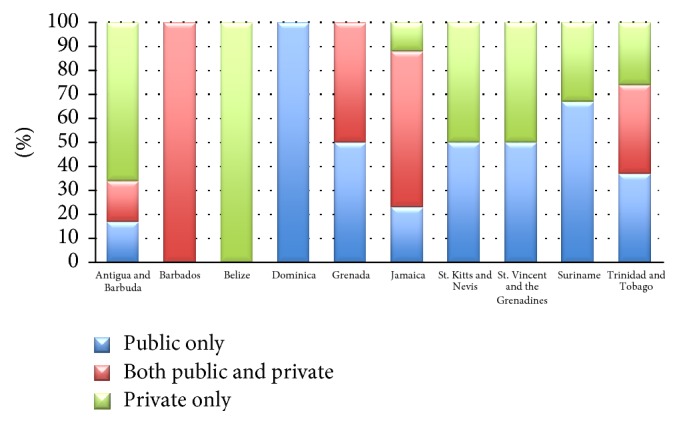
This illustrates the proportion of professionals working publicly or privately or offering services in both sectors.

Figure 5 illustrates the proportion of specialists working publicly, privately, or in both sectors. Belize and Antigua and Barbuda were the only countries in which more than 50% of the oncological workforce was concentrated in the private sector.

Table 2 indicates some of the frameworks necessary to support oncology services and the number of countries involved in each grouping. Only Trinidad and Tobago, Barbados, and Grenada reported the existence of a national cancer registry, with two other countries confirming that they were in the early stages of development. Sixty percent of the respondents indicated that hospital registries were available at some institutions. All treatment decisions were based on guidelines, and these tended to be international guidelines. In one country, Trinidad and Tobago, national guidelines were developed in 2015. In two of the ten countries, the respondents indicated that all treatment decisions were based on multidisciplinary team (MDT) discussions. In the remaining eight countries, the respondents reported that final management decisions were sometimes but not always based on MDT reviews. There were local health authority rules for the compounding of chemotherapy drugs in four nations. Radiation therapy was accessible in five countries, and most of these countries were formalizing legislation for the same.

## 4. Discussion

The increased demand for healthcare systems by the rising incidence and mortality related to cancer is substantial. There are many different factors which must be addressed to meet the challenge imposed on these countries adequately. Provision of adequate human resources, while not the only factor, is one of the major obstacles to providing the healthcare required. Therefore knowledge of the existing workforce is essential so that the deficiencies can be identified and plans can be made to address the shortfalls.

Estimating the required workforce is not a simple task, and no one assessment method is ideal. However, attempts have been made by various international organizations which seek to provide estimates on the potential numbers which can appropriately meet the required demands. As previously mentioned, in this study these estimates were taken from the IAEA with regard to radiation oncology needs and teletherapy machines, ASCO for medical oncology and ASH for hematology [2–5]. It can be argued that the recommendations made by the North American/European societies may not be directly applicable to another population grouping. They may, however, provide guidance on capacity needs. The results indicate that only one country (accounting for 10% of the countries reviewed) had an adequate provision of specialists offering radiation oncology services. The percentage for medical oncology was more favorable with 50% capacity achieved. In considering hematology services, only 60% of the countries evaluated had the required capacity. This concern over the lack of adequate oncology staffing is not a problem that is unique to the CARICOM region as other nations also face similar challenges [7–9]. One obvious way to address the problem would be to have individuals trained in the areas needed. This, however, is not without its challenges as outlined in a report produced by the WHO [10]. Training programs do not exist within the CARICOM region for radiation oncology. With regard to medical oncology and hematology, Jamaica has developed a training program in which physicians are taught management of both solid tumor and blood-based malignancies in a combined curriculum. Programs like this are appealing to regions which have staffing deficiencies as it allows for versatility in the provision of care despite the few specialists available. The same can be said for clinical oncology programs where individuals are trained in radiation therapy in addition to knowledge of systemic cytotoxic management. Like Jamaica, other Caribbean islands can consider developing similar programs. Trinidad and Tobago is in the process of gaining approval for the implementation of a similar program. In the absence of sufficient local/regional training programs, international training sites can be accessed. The study shows that this is already a common practice as many nationals have been pursuing training abroad, but the retention of these individuals is challenging. Skilled professionals are tempted by better remuneration packages which are offered in other regions. However, it should be noted that this is not the only obstacle to retaining staff. Nationals trained abroad as specialists are sometimes frustrated by the lack of equipment or supplies available to them on returning to their countries. These deficiencies hinder their ability to use their training optimally. Limitations such as the lack of oncology drugs or the absence of teletherapy machines prevent the physicians from fully applying their knowledge. In some instances, the education gained, therefore, remains theoretical and suffers from disuse as it cannot be put into practice. Obtaining and retaining adequate specialist numbers is critical in dealing with the growing burden of cancer care.

Multiple studies on the radiation oncologist density have shown that insufficient numbers are linked to increased mortality for some cancers [11–13]. Thus the fact that radiation oncology support is deficient in nine of the ten countries surveyed and absent in five of that grouping is troubling. The need for access to radiation therapy for treatment is further reinforced by the evidence that the optimal utilization rate for radiotherapy has been estimated at around 50% and thus approximately half of all cancer patients will require this treatment either as an adjunct to their primary management or as the sole therapy [14]. This is why associations such as the IAEA have reached out to some developing nations including the Caribbean with an aim to strengthen human capacities in radiation medicine. They have also developed training modules geared at teaching in the applied sciences for oncology through distance learning. These training modules did not confer specialist qualifications but were expected to be supplemental to formal training [15].

In hematology, the number employed in Jamaica appeared to be more than the required amount. It should be noted, however, that the majority of these specialists are dually trained to offer medical oncology services and that field remains an area which is understaffed. Thus reducing the hematology staff numbers will cause a concomitant decline in the provision of medical oncology care in that country. These hemato-oncologists in this setting were mainly locals. The same argument applies, in part, to Trinidad and Tobago where a similar situation occurs. The main difference is that a significant proportion of the hematology services in that country (50%) are provided by hematologists from Cuba who are solely trained in that specific specialty. These specialists were temporarily brought into the country and offer services covering both malignant and nonmalignant conditions. The training of local physicians in that field will reduce the need for foreign assistance. Antigua and Barbuda appear to be self-sufficient in its oncological staffing by adequately meeting and surpassing its recommended needs. Thus a case can be made for extending their services regionally to areas where these services are absent. In such a situation, one can make a case for specialists assisting in other islands as is the case for St. Kitts and Nevis which receives oncological support from a visiting hematologist. However, for these latter two countries, it was also found that greater than 50% of its oncological support was provided by specialists working outside of the public sector. The services, therefore, may only be accessible to a subset of the population who can afford to access care which is not provided by the state. It is imperative for all the countries involved that services be accessible to all patients regardless of their economic background.

However, providing the optimal number of physicians trained in hematology, medical oncology, and radiation oncology is not all that is required to address the oncology healthcare problem. The holistic management of the issue requires a broader overview. Human resource necessities must include surgeons, pathologists, radiologists, medical physicists, radiation therapists, dosimetrists, pharmacists, radiobiologists, palliative care specialists, and numerous other key support staff. Virtual MDT discussions with collaborating institutions in the Caribbean are practiced in a minority of treating facilities. However, the staff also need material and equipment to perform their duty optimally. Deficiencies in the access to radiation therapy machines, X-ray facilities, ultrasound, computed tomography, magnetic resonance imaging, flow cytometry, immunohistochemistry, molecular biology, and oncology drugs further cripple an already compromised situation. The staffing and resource limitations in these other areas must also be evaluated and would complement the information gleaned in a study such as this.

The amount of a nation's budget which should be allocated towards health has long been a topic of discussion [16]. The quantum of this funding which should be assigned to oncology is even harder to specify. Countries have to be selective when purchasing medication with a limited budget. Focusing on core drugs such as those outlined in the World Health Organization's (WHO) list of essential oncology drugs may be a reasonable place to start [17]. In addressing the problem of excessive drug costs, not only do smaller nations struggle with diminished budgets but the relatively low drug quantities required by their populations also significantly limits their bargaining power. Individual countries must therefore collaborate. Information such as that gleaned from the WHO multicountry regional pooled procurement of medicines report can be very instructive, and CARICOM as a region may benefit from pooling resources [18]. Some countries have already begun such collaborations, and others may follow. Accessing some drugs through frameworks such as the Pan-American Health Organization (PAHO) strategic drug fund can also be beneficial [19].

Ensuring that a country has all of the above serves only to treat the existing problem. We also need to be proactive in our approach, and that requires developing the appropriate screening programs for early diagnosis and patient education to help aid prevention and improve patient outcomes. However, data for the region are very limited [20]. Cancer registries are invaluable in gathering information which can help guide the development of public policy. The fact that only three countries admitted to the existence of a registry means that more work is required. Fortunately the region is getting support from organizations like the Caribbean Public Health Agency (CARPHA), the International Agency for Research on Cancer (IARC), the National Cancer Institute (NCI), the Centers for Disease Control and Prevention (CDC), and the North American Association of Central Cancer Registries (NAACCR) who are all collaborating to assist with developing and improving cancer registration [21]. Fortunately, many of the other countries have started institutional registries in the absence of a national registry, and this certainly is a positive start.

Government support is crucial in the implementation of many of the areas discussed above. Cancer is still not a notifiable disease in many countries. The problem is that this implementation is often a lengthy process but having the data to support it will certainly go a long way. Public policy and guidelines on mixing chemotherapeutic drugs and radiation legislation are needed, and since they were not available in most countries, this would have to be addressed.

Patient cancer organizations can be found on all the islands. Many of these groups are effective in disseminating information on cancer prevention and treatment. They are often funded by nongovernmental organizations and can be a valuable asset especially when resources are limited within a public sector framework.

## 5. Conclusion

The CARICOM group of full member states, and by extension of the rest of the Caribbean, continues to be an area with limited data. This paper is the first specifically to review the status of the current hematological and oncological physician workforce solely in that region. Providing a pathway for locals to be trained in oncology through scholarship funding will encourage an increase in the human resource capacity, but retention of qualified staff can be challenging. The development of locoregional training programs is preferable and will be of significant merit. The region as a whole needs to record and analyze regional cancer statistics, and the development of cancer registries will help to supply the relevant information. Developing countries need this data to help guide decisions on local protocol development in a setting where limited funding must be used prudently.

## Figures and Tables

**Table 1:** This shows the distribution of radiation therapy machines per country and the recommended number per population size based on recommendations by the International Atomic Energy Agency (1 machine per 180,00 inhabitants).

Country	Existing external beam radiation therapy machines			Recommended external beam radiation therapy machines
	Cobalt-60	LINAC	Total	
Antigua	-	1	1	1
Barbados	1	-	1	2
Jamaica	2	2	4	16
Suriname	-	2	2	3
Trinidad	1	3	4	8

**Table 2:** This indicates some of the frameworks necessary to support oncology services and the number of countries involved in each grouping.

	Yes	No	Sometimes/usually	Unknown
National cancer registry	3	7	-	-
Hospital registries	6	4	-	-
Treatment based solely on international guidelines	9	1	-	-
Multidisciplinary management	2	0	8	-
Patient cancer organizations	10	0	-	-
Local health authority rules for mixing cytotoxic drugs	4	6	-	-
Radiotherapy offered	5	5	-	-
Radiation legislation	2	7	-	1
